# Continuous short-term acclimation to moderate cold elicits cardioprotection in rats, and alters β-adrenergic signaling and immune status

**DOI:** 10.1038/s41598-023-44205-4

**Published:** 2023-10-25

**Authors:** Aneta Marvanova, Petr Kasik, Barbara Elsnicova, Veronika Tibenska, František Galatik, Daniela Hornikova, Veronika Zvolska, Pavel Vebr, Petr Vodicka, Lucie Hejnova, Petr Matous, Barbara Szeiff Bacova, Matus Sykora, Jiri Novotny, Jiri Neuzil, Frantisek Kolar, Olga Novakova, Jitka M. Zurmanova

**Affiliations:** 1 Faculty of Science, Department of Physiology, Charles University, Vinicna 7, 128 00 Prague 2, Czech Republic; 2 Institute of Animal Physiology and Genetics, Czech Academy of Sciences, Libechov, Czech Republic; 3 First Faculty of Medicine, Center for Advanced Preclinical Imaging (CAPI), Charles University, Prague, Czech Republic; 4 Centre of Experimental Medicine, Institute for Heart Research, Slovak Academy of Sciences, Bratislava, Slovak Republic; 5 Institute of Biotechnology, Czech Academy of Sciences, Prague-West, Czech Republic; 6 School of Pharmacy and Medical Science, Griffith University, Southport, QLD Australia; 7 Institute of Physiology, Czech Academy of Sciences, Prague, Czech Republic

**Keywords:** Physiology, Cardiology, Diseases

## Abstract

Moderate cold acclimation (MCA) is a non-invasive intervention mitigating effects of various pathological conditions including myocardial infarction. We aim to determine the shortest cardioprotective regimen of MCA and the response of β1/2/3-adrenoceptors (β-AR), its downstream signaling, and inflammatory status, which play a role in cell-survival during myocardial infarction. Adult male Wistar rats were acclimated (9 °C, 1–3-10 days). Infarct size, echocardiography, western blotting, ELISA, mitochondrial respirometry, receptor binding assay, and quantitative immunofluorescence microscopy were carried out on left ventricular myocardium and brown adipose tissue (BAT). MultiPlex analysis of cytokines and chemokines in serum was accomplished. We found that short-term MCA reduced myocardial infarction, improved resistance of mitochondria to Ca^2+^-overload, and downregulated β1-ARs. The β2-ARs/protein kinase B/Akt were attenuated while β3-ARs translocated on the T-tubular system suggesting its activation. Protein kinase G (PKG) translocated to sarcoplasmic reticulum and phosphorylation of AMPK^Thr172^ increased after 10 days. Principal component analysis revealed a significant shift in cytokine/chemokine serum levels on day 10 of acclimation, which corresponds to maturation of BAT. In conclusion, short-term MCA increases heart resilience to ischemia without any negative side effects such as hypertension or hypertrophy. Cold-elicited cardioprotection is accompanied by β1/2-AR desensitization, activation of the β3-AR/PKG/AMPK pathways, and an immunomodulatory effect.

## Introduction

Despite recent progress in biomedicine, ischemic heart disease remains the most common cause of death and comorbidity worldwide. This grim picture stems from the fact that many promising therapeutic approaches demonstrated in animal models have failed in clinical trials^1,2^. Cold acclimation (CA) has so far been successfully studied also in the context of improving health complications in the metabolic syndrome^3,4^, which includes chronic inflammation and oxidative stress. In humans, chronic CA significantly increases antioxidant capacity in blood serum and decreases homocysteine levels, suggesting a beneficial effect on the cardiovascular system^5^. Nevertheless, hypertension and left ventricle hypertrophy and other detrimental effects were repeatedly documented in animals exposed to severe cold^6^. Clinical trials using moderate cold in treatment of obesity and diabetes have been reported^7–9^. Since the beneficial effect of CA depends on the intensity of the cold and the mode of adaptation regarding the given organism, it is necessary to understand when and under which conditions the protective effect of CA occurs and what is its molecular basis.

We have recently shown that an appropriate moderate cold acclimation (MCA) regimen presents a promising cardioprotective intervention. Chronic gradual MCA (8 ºC, 5 weeks) reduced the extent of myocardial infarction without negative side effects such as hypertension and hypertrophy. This model also improved mitochondrial resistance to Ca^2+^-overload, and preserved the β1-adrenergic receptor (β-AR) function^10,11^.

A β-AR signaling in the heart controls cardiac function under both physiological and pathophysiological conditions^12^ and, importantly, it is a powerful regulator of immune response in context of ischemic injury^13–15^. The β1-ARs are coupled to G-stimulatory (Gs) proteins, which in turn stimulate protein kinase A (PKA) via hormone-stimulated cAMP formation by adenylyl cyclase^16^. Sustained activation of β1-AR signaling is deleterious and can promote apoptosis of cardiomyocytes, which occurs during hypertension or chronic heart pathologies, leading to progressive hypertrophy and culminating in heart failure^17,18^. It is generally accepted that minor β2/3-AR subtypes in the heart also stimulate adenylyl cyclase activity. However, both β2/3-ARs can also couple to G-inhibitory (Gi) proteins to attenuate β1-AR hyperactivation^19^. In this framework, activation of Gi-coupled signaling pathways, β2-ARs/protein kinase B (Akt) and β3-ARs/protein kinase G (PKG) have been confirmed as cardioprotective under certain stress conditions such as chronic hypoxia^20^, exercise training^21^, and the recently demonstrated recovery phase of chronic CA^10^. Stimulation of β2/3-ARs mediates protection against hypertrophic or fibrotic remodeling^19,22^. Recently, β3-AR coupling to AMPK, a key metabolic sensor, has been identified as a cardioprotective mechanism that preserves the downstream autophagy process^23^.

CA is a highly complex adaptive process mediated via whole body neuroendocrine stimuli of the adrenergic system, reinforcing the thyroidal hormones action and leading to formation of brown adipose tissue (BAT)^24^. The β1/3-AR receptor/cAMP/PKA and AMP-activated protein kinase (AMPK) pathways play a crucial role in BAT formation. Both pathways control non-shivering thermogenesis of BAT via increased glucose uptake, mitochondrial biogenesis, fatty acid metabolism, and upregulation of uncoupling protein-1 (UCP1)^25^. Besides heat production, mature brown adipocytes are characterized by secretory function, releasing several protective bioactive molecules (batokines) into the bloodstream. The batokine fibroblast growth factor 21 (FGF21) serves as a marker of BAT maturation, and its plasma levels in humans are associated with cold-induced BAT activity^26^. It was proposed that UCP1 and BAT-released FGF21 target the heart to exert cardioprotective effects^27^. High metabolic activity and dissipation of energy is a promising intervention for diabetic patients even during short-term MCA^3^.

The major goal of the present study is to find out the minimum duration of MCA that improves cardiac tolerance to acute ischemia/reperfusion (I/R) injury. And subsequently, to explore a series of plausibly connected events that may explain MCA-induced cardioprotection as a basis for future mechanistic studies using reductionist approaches. Thus, we asked the following questions. (1) What is the role of β1/2/3-AR downstream signaling and mitochondria in the MCA-elicited cardioprotection? (2) Is there a role for circulating batokines (FGF21, IL-6) in the cardioprotection? (3) How does the novel cardioprotective regimen of MCA affect the inflammatory status of the heart and the whole organism? Answers to these questions should provide a new insight into the complexity of cardioprotective mechanisms induced by short-term MCA.

## Results

### Optimization of cold acclimation protocol and its safety profile

In the present study, we tested whether short-term exposure to cold results in an improvement of cardiac ischemic tolerance. Based on our preliminary data, we chose 1, 3 and 10 days of cold exposure at a temperature below the threshold of shivering thermogenesis and (9 ± 1 °C)^11,28^ and characterized the time course of BAT activation (mitochondrial biogenesis, AMPK activation, UCP1, and FGF21 levels), a possible release of batokines into the circulation and the cytokine profile in the blood serum, as well as myocardial responses, in order to reveal potential players in cold-elicited cardioprotection.

The effect of short-term MCA on basic parameters is documented in Table 1. Data show that the weight of BAT and the BAT/body weight ratio, a marker of the cold-acclimated phenotype, increased by 54% and 60%, respectively, after 10 days. The weight of adrenal glands, a marker of cold stress, did not change significantly during the 10 days of acclimation. The lack of a change in adrenal gland/body weight supports the notion of well tolerable (moderate) cold stress stimuli. Concerning the cardiac and body parameters, the MCA did not affect the body weight, body temperature, heart weight or the heart/body weight ratio, ruling out hypothermia, hypertension, and myocardial hypertrophy. Also, it did not affect the heart rate and the mean arterial blood pressure during the I/R (Table 2). The data above indicate that short-term moderate cold acclimation causes a cold-adaptive phenotype on day 10 without any negative side effects within the tested parameters.

**Table 1:** Body weight (BW); brown adipose tissue (BAT); rectal temperature (RT); heart weight (HW); left ventricle (LV); ventricular septum (S); right ventricle (RV); adrenal glands (ADG); (n = 8); values are means ± S.D.; **p < 0.01, ***p < 0.001 vs. control. One-way ANOVA with Dunnett’s multiple comparison test.

	Control	1 day	3 days	10 days
N	8	8	8	8
BW (g)	385 ± 16	363 ± 13	356 ± 21	370 ± 30
BAT (mg)	257 ± 47	270 ± 57	266 ± 86	396 ± 71**
BAT/BW	0.67 ± 0.12	0.74 ± 0.16	0.74 ± 0.23	1.07 ± 0.11***
RT (°C)	37.0 ± 0.23	36.9 ± 0.54	36.7 ± 0.27	36.9 ± 0.31
HW (mg)	1016 ± 83	1059 ± 79	1018 ± 58	1030 ± 86
LV + S/BW	2.07 ± 0.23	2.21 ± 0.26	2.24 ± 0.08	2.16 ± 0.13
RV/BW	0.57 ± 0.05	0.57 ± 0.06	0.61 ± 0.07	0.62 ± 0.07
HW/BW	2.64 ± 0.22	2.78 ± 0.26	2.86 ± 0.11	2.79 ± 0.12
ADG/BW	0.15 ± 0.03	0.16 ± 0.04	0.18 ± 0.02	0.18 ± 0.03

**Table 2:** Heart rate and mean arterial blood pressure (n = 8–12); values are means ± S.D.; *p < 0.05, vs. Control; two-way ANOVA with Dunnett’s multiple comparison test (the effect of cold acclimation) and two-way ANOVA with Šidák’s multiple comparison test (the effect of I/R).

Heart rate, beats/min				
	Control	1 day	3 days	10 days
N	8	12	12	12
Baseline	390 ± 17	403 ± 20	398 ± 22	382 ± 30
Ischemia	384 ± 33	395 ± 23	382 ± 31	380 ± 33
Reperfusion	375 ± 29	395 ± 30	385 ± 36	382 ± 35
Blood pressure, mmHg				
Baseline	91 ± 24	83 ± 18	92 ± 16	70 ± 18*
Ischemia	77 ± 19	73 ± 11	88 ± 18	74 ± 23
Reperfusion	76 ± 16	70 ± 12	84 ± 17	70 ± 17

### Characterization of BAT maturation during moderate cold acclimation

**Figure 1:**
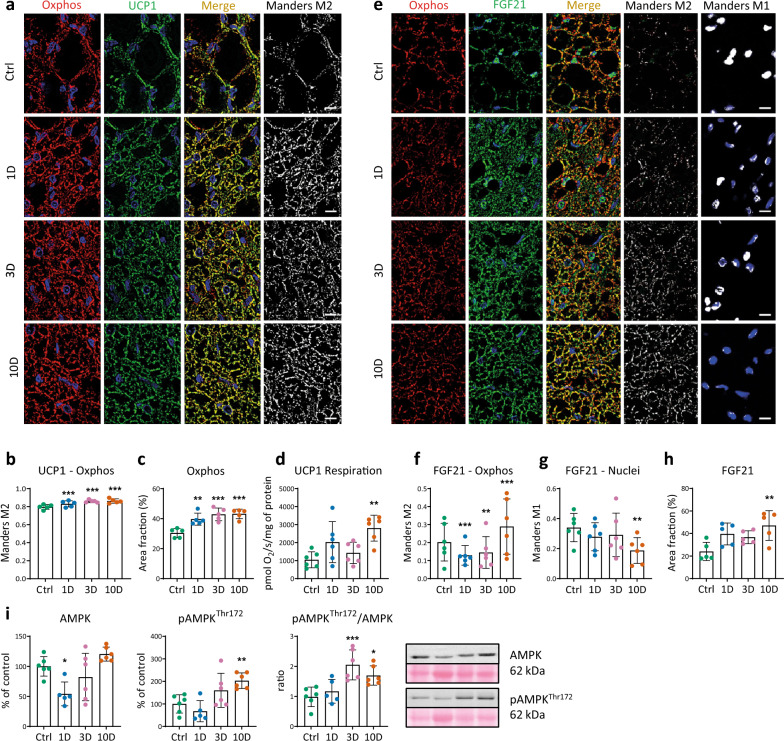
Characterization of brown adipose tissue (BAT) maturation during cold exposure (9 ± 1 °C) for 1–3–10 days (1D, 3D, 10D) and in control rats (Ctrl). (**a**) Representative images of BAT mitochondria (red color; anti-OXPHOS Abs) and the uncoupling protein UCP1 (green color), blue color indicates nuclear DAPI staining. Merged column shows colocalization of UCP1 with OXPHOS (yellow-orange color), black and white images show corresponding colocalized pixels (Mander’s M2 correlation coefficient), quantified in the graph (**b**) (n = 5). (**c**) Mitochondrial density represented by the area fraction shown as red signal. (**d**) UCP-dependent respiration of isolated mitochondria (n = 5–6). (**e**) Representative image of FGF21 (green color) and mitochondria (red color). Merged column shows colocalization of FGF21 with mitochondria (yellow-orange, Mander’s M2) and with nuclei (blue-green, Mander’s M1), respectively. Black and white images show corresponding colocalized pixels quantified in graphs (**f,g**). (**h**) FGF21 density represented by area fraction (%) of green positive signal. **i.** Relative protein level of total AMPK and p-AMPK^Thr172^ obtained by western blots, and the p-AMPK^Thr172^/AMPK ratio (n = 5–6). Data presented in graphs were analyzed by One-way ANOVA with Dunnett’s multiple comparison test. Values are means ± SD; *p < 0.05, **p < 0.01, ***p < 0.001 vs. control. Scale bar 10 μm.

To determine the activation and maturation of BAT in the early stages of MCA, we analyzed the morphological changes, and expression and distribution of specific markers of BAT maturation. We found a 30% increase in the BAT mitochondrial mass, expressed as fractional area of cryosections, already after 1 day of MCA, and an increase of 40% on days 3 and 10 (Fig. 1a, c). Co-localization of UCP1 with mitochondria increased slightly after 1 day and elevated by 10% on days 3 and 10 (Fig. 1a, b), while mitochondrial UCP1-dependent respiration was markedly elevated after 10 days (by 20%) (Fig. 1d). Immunofluorescence analysis of BAT cryosections revealed that the level of FGF21, the main batokine produced during BAT maturation, increased after 10 days (Fig. 1e, h). Similarly, FGF21 colocalization with the mitochondrial compartment increased after 10 days (Fig. 1e, f), while it decreased after 1 and 3 days of MCA. Nuclear localization of FGF21 gradually decreased with its increasing localization in mitochondria (Fig. 1e, f, g). The pAMPK^Thr172^/AMPK ratio, reflecting the metabolic activity of BAT, was elevated by 108% after 3 days and was elevated by 72% on day 10 of MCA (Fig. 1i).

### Myocardial ischemic tolerance during moderate cold acclimation

**Figure 2:**
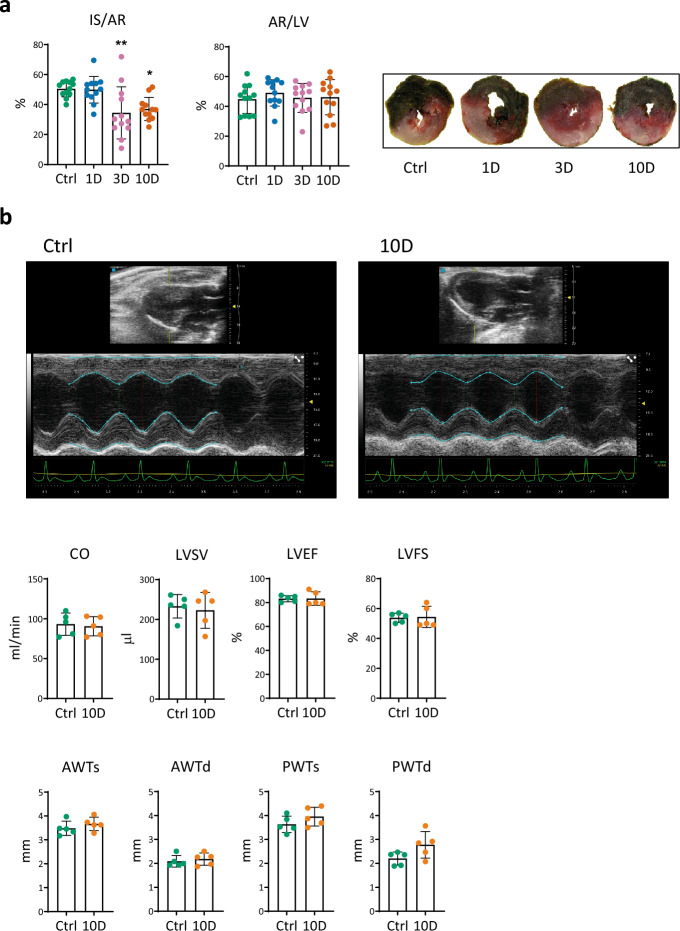
The effect of short-term cold acclimation on myocardial sensitivity to ischemia/reperfusion injury and cardiac function. (**a**) The extent of myocardial infarction in vivo in control rats (Ctrl) and those exposed to 9 ± 1 °C for 1–3-10 days (1D, 3D, 10D) and respective representative images. Infarct size (IS) was expressed as a percentage of area at risk (AR); AR was normalized to the cross-section area of left ventricle (LV) (n = 12). (**b**) Echocardiographic data of rats prior and after 10D of the exposure. Representative M-mode tracing of the left ventricle and following echocardiographic measurements; the cardiac output (CO), left ventricular stroke volume (LVSV), ejection fraction (LVEF), fractional shortening (LVFS) and systolic/diastolic anterior (AWTs/d) and posterior (PWTs/d) left ventricular wall thickness were evaluated by the Vevo LAB software. Data presented in graphs were analyzed by One-way ANOVA with Dunnett’s multiple comparison test. Values are means ± SD; *p < 0.05; **p < 0.01 vs. Ctrl.

Analysis of the extent of I/R injury revealed that 3- and 10-day moderate cold exposure reduced myocardial infarction to 34% and 37% of the area at risk (AR), respectively, compared with 50% in the control group, while 1 day of cold exposure had no effect (Fig. 2a*, *left). The average ratio of normalized AR to the left ventricle (AR/LV) reached 45—49% and did not differ between the groups (Fig. 2a*, *right). Increased resilience of isolated cardiomyocytes to hypoxia/reoxygenation elicited by 10 day of MCA was also observed (data not shown). Echocardiography did not reveal any differences between the groups, and the unchanged diameters of the anterior and posterior LV walls excluded cold-elicited hypertrophy after 10 days of cold exposure (Fig. 2b*)*.

### Mitochondria and AMPK in cold-elicited cardioprotection

**Figure 3:**
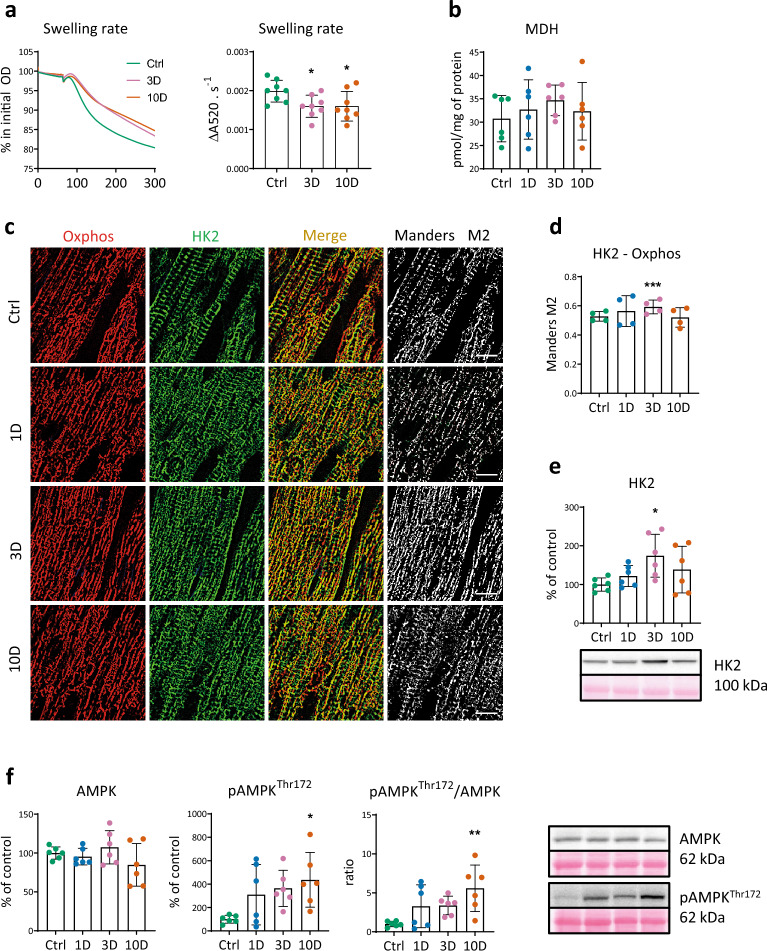
Effect of short-term cold acclimation on mitochondrial resilience to Ca^2+^ overload, oxidative stress marker (malondialdehyde, MDH), hexokinase 2 (HK2) translocation to mitochondria and AMPK in the left ventricle (LV) of control rats (Ctrl) and those exposed to 9 ± 1 °C for 1–3-10 days (1D, 3D, 10D). (**a**) Representative recordings of induced mitochondrial maximal swelling rate at 200 μM Ca^2+^ (left) expressed as the change of absorbance (DA) per 1 s (right) (n = 6). (**b**) Concentration of malondialdehyde (MDH) in LV homogenates (n = 6). (**c**) Representative images documenting mitochondrial compartment (red color; anti-OXPHOS Abs) and HK2 isoform (green color). Merged column represents respective co-localization (yellow-orange color), black and white images show corresponding co-localized pixels. (**d**) Quantification of the colocalizations Mander’s M2 coefficients. Scale bars, 10 µm, (n = 4–5; five ROIs for each). (**e**) Level of HK2 protein in mitochondrial fraction expressed as the percentage of Ctrl (n = 6). (**f**) Relative levels of AMPK and p-AMPK^Thr172^ proteins in homogenate, and the p-AMPK^Thr172^/AMPK ratio (n = 6). Data presented in graphs were analyzed by One-way ANOVA with Dunnett’s multiple comparison test. Values are means ± SD; *p < 0.05,**p < 0.01, ***p < 0.001 vs. Ctrl.

To uncover a possible role of MPT pore in the cardioprotective mechanism, we tested the maximal mitochondrial swelling rate using 200 µM Ca^2+^ and found that it was reduced in both ‘protected’ groups: on day 3 by 19% and day 10 by 21% compared to the control group (Fig. 3a). The concentration of malondialdehyde in the LV heart homogenate did not show significant changes during the MCA, which excludes oxidative stress development during its acute phase (Fig. 3b). Using quantitative immunofluorescence microscopy and western blotting of mitochondrial fractions, we examined translocation of the HK2 isoform to the outer mitochondrial membrane, which is known to prevent MPT pore opening. Co-localization of HK2 increased after 3 days of MCA (Fig. 3c, d), which was confirmed by elevated level of HK2 protein in the mitochondrial fraction (Fig. 3e). Both values returned to the control level after 10 days of MCA (Fig. 3c–e). Importantly, phosphorylation of p-AMPK^Thr172^ increased after 10 days of MCA as well as the p-AMPK^Thr172^/AMPK ratio (Fig. 3f), suggesting stimulation of the pleiotropic role of AMPK in the cardioprotection of cold-acclimated rats.

### Myocardial β-adrenergic signaling during moderate cold acclimation

**Figure 4:**
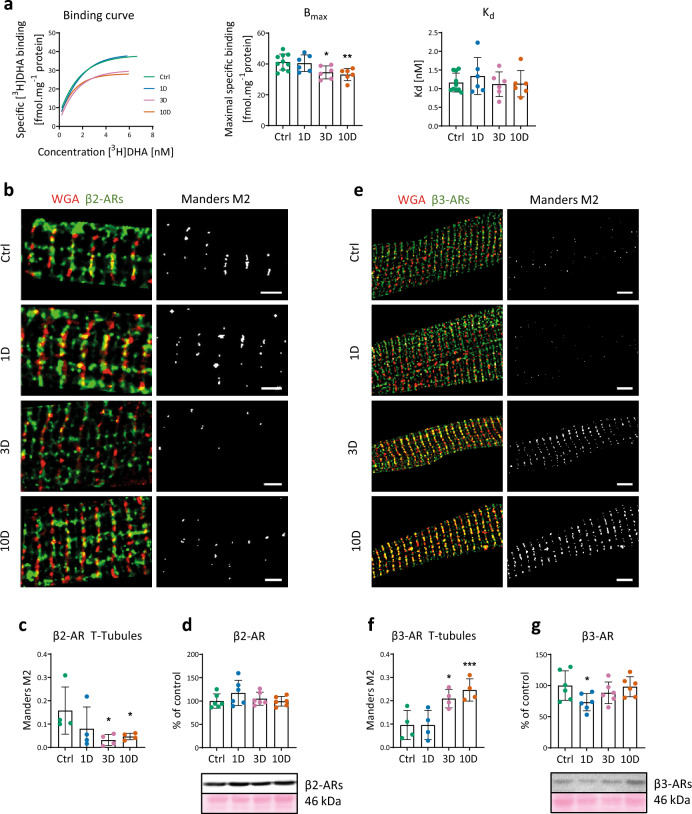
Effect of short-term cold acclimation (MCA) on the total number of β-adrenergic receptors (β-ARs) and their subcellular distribution on day 1–3–10 days of MCA (1D, 3D, 10D) and in control rats (Ctrl). (**a**) Representative saturation binding curves constructed by assessing binding of [3H]-dihydroalprenolol ([3H]-DHA) to myocardial crude membranes using increasing concentration of the radioligand (three saturation binding experiments were performed in triplicate, and the graph shows typical saturation binding curves), β-AR maximal binding capacity (Bmax), and receptor affinity (Kd) (n = 6). (**b,e**) Representative images documenting colocalization of β2- and β3-ARs, respectively (green color) with the T-tubular system stained by WGA (red color). Black and white images represent colocalized pixels. Scale bars, 2 µm (**b**) and 5 µm (**e**). (**c,f**) Quantification of the colocalization with the T-tubular compartment was calculated as Mander’s M2 coefficient (n = 4). (**d,g**) Relative protein levels of β2- and β3-ARs in the crude membrane fraction (n = 6). Data shown in the graphs were analyzed by One-way ANOVA with Dunnett’s multiple comparison test. Values are means ± SD; *p < 0.05, **p < 0.01, ***p < 0.001 vs. Ctrl.

The balance in the isoforms of β1/2/3-ARs and their downstream pathways play an important role in the cardioprotective phenotype. Therefore, we analyzed the total number and affinity of β-ARs using a specific binding assay, as well as the expression and localization of β2- and β3-ARs in the crude membrane fraction. After 3 and 10 days of MCA, the total number of myocardial β-ARs was 16% and 18% lower, respectively, compared to controls (Fig. 4a), reflecting a decline in major β1-ARs. Immunofluorescence analysis revealed changes in the localization of both β2- and β3-AR proteins after MCA (Fig. 4b, e). Despite the very low T-tubular occupancy by β2-ARs in the controls, β2-ARs decreased even more on days 3 and 10 (Fig. 4b, c). Conversely, the association of β2-ARs with the surface sarcolemma increased after 3 days (not shown). The occupancy of T-tubules by β3-ARs increased after 3 and 10 days, and their localization within the sarcolemmal compartment showed no significant changes during MCA (Fig. 4e, f). On the other hand, levels of β2- and β3-AR proteins, assessed by western blotting, were unchanged in the crude membrane fraction (Fig. 4d, g).

**Figure 5:**
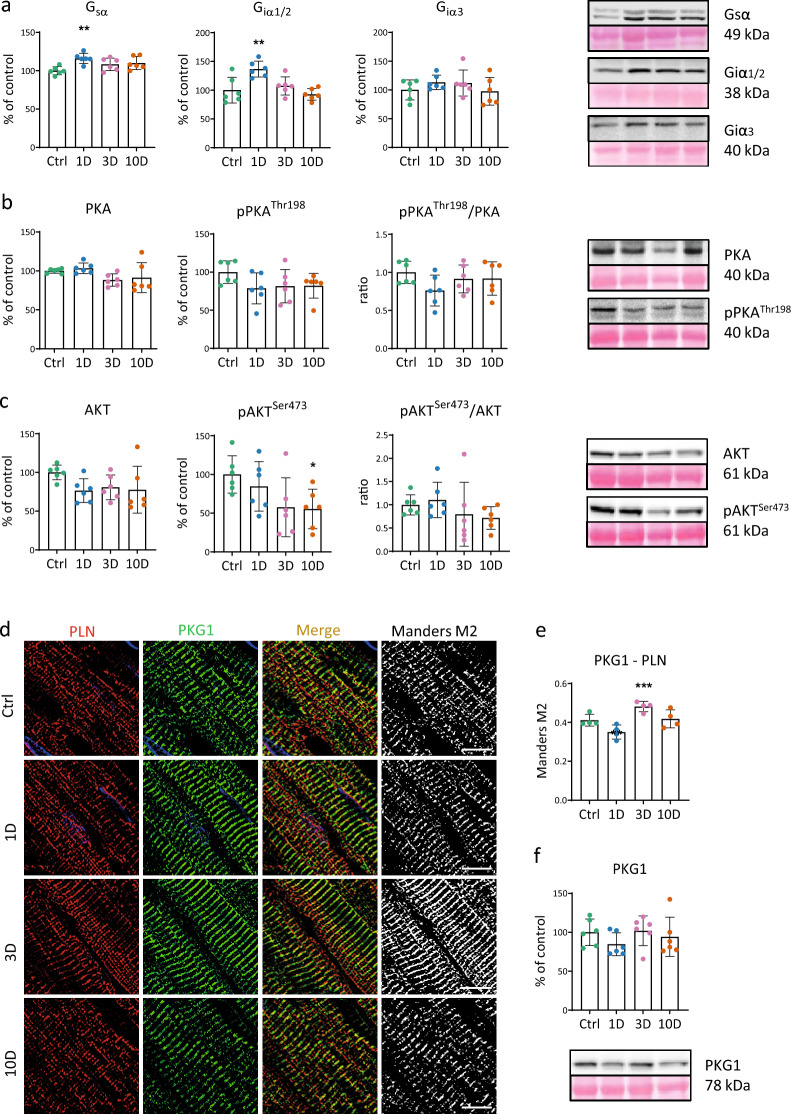
Effect of short-term cold acclimation (CA) on the β1/2/3-ARs-signalling pathways. (**a**) Relative G-protein (Gsα, Giα1/2, and Giα3) levels in a crude membrane fraction expressed as percentage on day 1–3-10 days (1D, 3D, 10D) of CA, and in control rats (Ctrl) (n = 6). (**b**) Relative protein levels of protein kinase A (PKA), p-PKAThr198, and p-PKA^Thr198^/PKA ratio (n = 6). (**c**) Relative protein levels of protein kinase B/Akt (Akt), p-Akt^Ser473^, and p-Akt^Ser473^/Akt ratio (n = 6). (**d**) Representative images documenting localization of phospholamban (PLN, red color) and protein kinase G (PKG1, green color). Merged column represents co-localization (yellow-orange color), black and white images show corresponding co-localized pixels. Scale bar 10 μm. (**e**) Quantification of colocalization with the T-tubular compartment were calculated as a Mander’s M2 coefficient (n = 4). (**f**) Relative protein level of PKG1 in the crude membrane fraction (n = 6). Data shown in the graphs were analyzed by One-way ANOVA with Dunnett’s multiple comparison test. Values are means ± SD; *p = 0.05, **p < 0.01, ***p < 0.001 vs. Ctrl.

Expression of G_sα_ and G_iα1/2_ in crude membrane fractions increased significantly only after 1 day, while G_iα3_ was not affected (Fig. 5a). Expression and phosphorylation of PKA, a component of the downstream β1/β2-ARs/G_sα_ pathway, were not significantly altered by MCA (Fig. 5b). Expression of total PKB/Akt, a downstream kinase of β2/β3-ARs/G_i_ pathways was not significantly altered. However, the phosphorylation of Akt at Ser473 residue declined after 10 days (p = 0.05) (Fig. 5c) suggesting suppression of its activity. Regarding PKG, a downstream kinase of β3-ARs/G_iα1/2_, we detected a translocation of PKG1 to the membrane of the sarcoplasmic reticulum identified by staining with anti-phospholamban antibody after 3 days of MCA (Fig. 5d, e), while its expression was not altered (Fig. 5f).

### FGF21 and inflammatory markers during moderate cold acclimation

**Figure 6:**
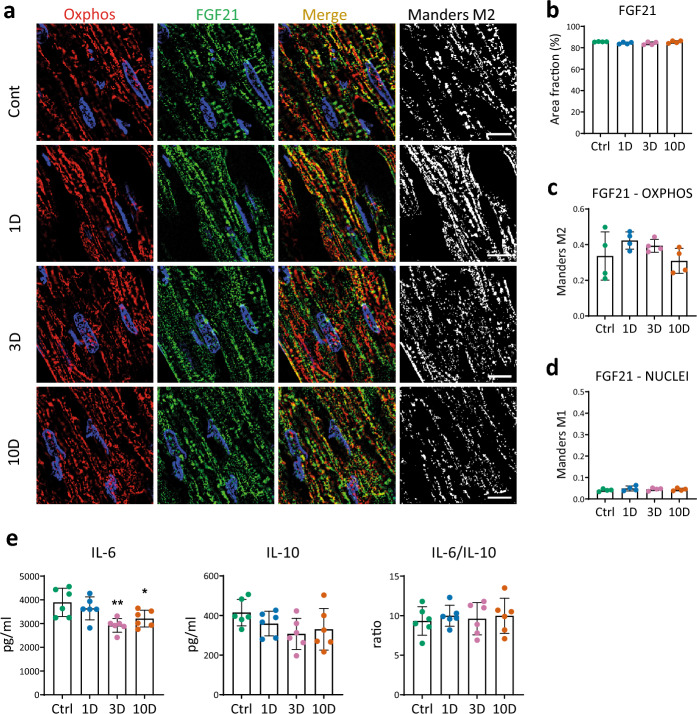
Inflammatory markers and expression of FGF21 in the left ventricle (LV) myocardium. (**a**) Representative images documenting spatial expression of FGF21 in control rats (Ctrl) and these exposed to 9 ± 1 °C for 1–3–10 days (1D, 3D, 10D). Co-localization of FGF21 (green) with the mitochondrial compartment (red; anti-OXPHOS IgGs) is shown in yellow-orange. Black and white images show corresponding colocalized pixels. Blue color indicates DAPI staining. (**b**) FGF21 density represented by the area fraction (%) of green positive signal. (**c**) Quantification of FGF21 colocalization with mitochondria was calculated as a Mander’s M2 coefficient. (**d**) Quantification of FGF21 colocalization with nuclei was calculated as a Mander’s M1 coefficient (n = 4; five ROIs for each sample. (**e**) Concentration of pro- and anti- inflammatory cytokines (IL-6, IL-10) and their ratio in LV homogenates. Data presented in graphs were analyzed by One-way ANOVA with Dunnett’s multiple comparison test, values are means ± SD; *p < 0.05, **p < 0.01, ***p < 0.001 vs. Ctrl. Scale bars 10 µm.

Regarding the heart, the spatial expression and distribution of the batokine FGF21, one of the cytokine candidates for cardioprotection, were quantified in longitudinal LV sections by immunofluorescence (Fig. 6a) similarly as shown for BAT in Fig. 1. The area fraction of FGF21 reflecting its spatial expression did not differ between the groups (Fig. 6a, b). However, we observed differences in the subcellular distribution FGF21 in the heart and in BAT. In BAT, Mander’s correlation coefficients M1 and M2 documented altered colocalization of FGF21 with mitochondria and nuclei during MCA, while in the heart FGF21 colocalized with mitochondria (Fig. 6a, c) but not with nuclei (Fig. 6a, d). Colocalization with mitochondria did not differ between the groups (Fig. 6c).

ELISA analysis revealed that the concentration of the pro-inflammatory cytokine IL-6 significantly decreased on day 3 and remained decreased on day 10 of MCA in LV homogenates. The anti-inflammatory cytokine IL-10 did not change significantly and only tended to decrease resulting in the IL-6/IL-10 ratio remaining unaltered (Fig. 6e). This suggests a moderate anti-inflammatory effect of MCA in the LV myocardium.

### Serum concentration of cytokines

**Figure 7:**
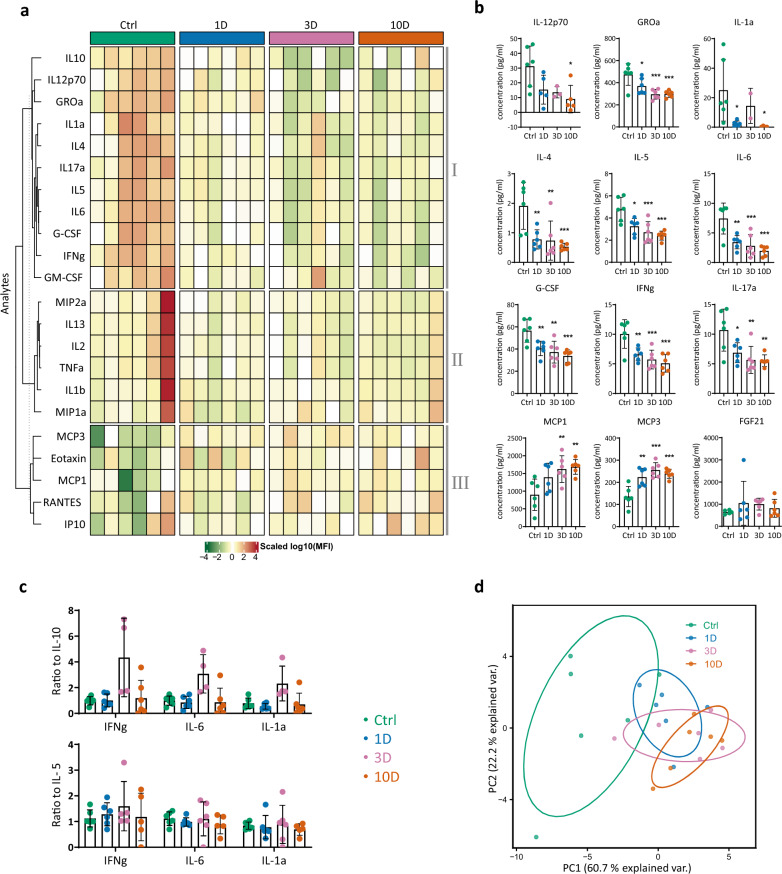
Multivariate analysis of cytokines and chemokines in arterial blood serum samples by Multiplex analysis from control rats (Ctrl) and rats exposed to 9 ± 1 °C for 1–3–10 days (1D, 3D, 10D). (**a**) The heatmap documents changes in the concentrations of 14 cytokines (G-CSF, GM-CSF, IFNg, IL-1α, IL-1β, IL-2, IL-4, IL-5, IL-6, IL-10, IL12p70, IL-13, IL-17A, TNFα) and 8 chemokines (Eotaxin, GROα, IP-10, MCP-1, MCP-3, MIP-1α, MIP-2, RANTES) expressed as scaled log10 (MFI) in three panels (I, II, III). (**b**) Significant changes in the analytes elicited by moderate cold exposure. (**c**) Inflammatory indexes calculated as the ratio of generally accepted pro-inflammatory IFNγ, IL-1α and IL-6, and anti-inflammatory IL-10 and IL-5 cytokines calculated from normalized values. (**d**) Multivariate analysis (PCA) of all analytes showing separation of the four experimental groups (Ctrl, green; 1D, blue; 3D, pink; 10D, orange) by first two principal components (PC1, PC2). The points represent individual samples, and ellipses 68% confidence interval for each group. The points represent individual samples, values are means ± SD; *p < 0.05, **p < 0.01, ***p < 0.001 vs. Ctrl. One-way ANOVA with Dunnett’s multiple comparison test.

Next, we evaluated number of cytokines level in the serum. The heatmap in Fig. 7a shows changes in concentrations of 14 cytokines (G-CSF, GM-CSF, IFNγ, IL-1α, IL-1β, IL-2, IL-4, IL-5, IL-6, IL-10, IL-12p70, IL-13, IL-17A, TNFα) and 8 chemokines (Eotaxin, GROα, IP-10, MCP-1, MCP-3, MIP-1α, MIP-2, RANTES) expressed as scaled log10 (MFI). MCA elicited gradual changes in concentration of most tested cytokines that can be divided into 3 main clusters. The first cluster (I) on the heatmap includes 1 chemokine (GROα) and most of the cytokines that declined during MCA (GROα, G-CSF, GM-CSF, IFNγ, IL-1α, IL-4, IL-5, IL-6, IL-10, IL-12p70, IL-17A). The significant differences are shown in Fig. 7b. The middle cluster (II) on the heatmap includes 2 chemokines (MIP1a, MIP2) and 4 cytokines (IL-1β, IL-2, IL-13, TNFα), showing a moderate trend to decrease during the MCA process. The third cluster (III) manifests a trend of increased concentration, comprising only chemokines (eotaxin, IP-10, MCP-1, MCP-3, RANTES) of which only MCP-1 and MCP-3 reached significance (Fig. 7a, b).

As the first cluster (A) on the heatmap shows decline of both pro- and anti-inflammatory cytokines, we calculated the inflammatory index as the ratio of IFNγ, IL-1α, IL-6 to each of IL-10 and IL-5 from normalized values (Fig. 7c). Changes of the inflammatory index were not significant across the groups. Moreover, all inflammatory indexes tend to increase on day 3 when IL-10 was used as a denominator (Fig. 7c). PCA analysis shown in Fig. 7d clearly separates two clusters of the control (green) and day 10 group (orange), while the day 1 and day 3 groups overlap with other groups, manifesting a transient state of the immune response to cold during the early period of acclimation (Fig. 7d). The presented data document a significant shift to lower levels of key cytokines on day 10 of MCA, while the balance between pro- and anti-inflammatory cytokines remains unchanged between controls and experimental groups.

## Discussion

Repeated exposure to mild cold has been presented in a few clinical trials as a successful therapeutic intervention in type II diabetes and obesity^7–9^. We have recently demonstrated an infarct size-limiting effect of chronic moderate cold exposure for 5 weeks at 8 ± 1 °C in rats, which persisted at least 2 weeks after the animals returned to control temperature^10,11^. Understanding the cellular and molecular processes during the early development of cold-elicited cardioprotective phenotype is important, especially considering potential therapeutic application. This requires a sensitive setting of the cold intensity and regimen considering an individual's health and constitution. Therefore, we aimed to determine whether the period of moderate acclimation required for cardioprotection could be further shortened and, if so, to characterize the relevant model.

In the present study we demonstrate for the first time that moderate cold acclimation achieved through exposure to 9 ± 1 °C for 3 and 10 days, but not for one day, suffices to reduce the infarct size, which distinguishes MCA from the effects of classic pre- or post-conditioning^10,11^. Both protective stages of MCA were accompanied by an increased mitochondrial resistance to Ca^2+-^overload followed by β1-ARs desensitization, and increased compartmentalization of β3-ARs within transverse T-tubules. After 10 days of MCA, we observed a noticeable decrease in β2-ARs within the T-tubular system and in the phosphorylation of its downstream Akt^Ser473^ kinase. However, the expression and phosphorylation levels of PKA were not significantly altered. We propose that a fully acclimated phenotype is achieved under these conditions on day 10, as we observed BAT maturation at this time point of MCA, but not on day 3. The given phenotype involves AMPK activation in the heart, and we also found clustering of serum cytokines obtained by PCA analysis. For the specific signaling in cardiac tissue on day 3, MCA led to beneficial co-localizations of PKG with phospholamban on the sarcoplasmic reticulum and of HK2 with mitochondrial outer membrane. An anti-inflammatory effect was also evident in the LV homogenate. We did not observe any of the negative side effects reported for more severe cold conditions in rats (below 5 °C), such as systemic hypertension, LV hypertrophy, hypothermia, or adrenal gland hyperactivation which were described in previous studies^29^. The presented findings highlight the importance of determining the appropriate regimen of cold exposure with respect to the organism in order to achieve its beneficial effect. The duration of the protective action of short-term MCA after its cessation remains to be determined.

### Adrenergic signaling, mitochondria, and AMPK recruitment during MCA

Our data suggest that a key point of the MCA-elicited cardioprotection is the enhanced mitochondrial resilience resulting from the shift in adrenergic signaling (β1-AR desensitization and enhancement of β3-AR in the T-tubular system) and AMPK activation. Trappanese et al. documented a link between β1-AR blockade and β3-AR coupling with nitric oxide-linked cGMP signaling^30^. Functional β3-ARs localize exclusively within the transverse T-tubules of healthy rat cardiomyocytes, and its dysregulation occurs in the failing heart^31^. Binding of catecholamines to β3-ARs induces negative inotropic and positive lusitropic effects via the inhibitory pathway of Gi/cGMP/PKG^32,33^ and via the control of Ca^2+^ handling^34^. β3-AR/PKG-mediated moderation of Ca^2+^ transient currents can occur via NOS-dependent inhibition of L-type channels, attenuating excitation–contraction coupling^35^. Moreover, PKG-mediated phosphorylation of phospholamban improves Ca^2+^ uptake during cardiac myocyte relaxation^36^. Interestingly, while total Akt tended to decline, p-Akt^Ser473^ decreased significantly in the fully acclimated heart after 10 days of MCA. Recently, we reported that chronic cold exposure (5 weeks) did not alter Akt signaling, whereas the cardioprotection observed after 2 weeks of return to normothermic conditions required the activation of the Akt signaling pathway^10^. Concerning mitochondrial protection, we show here that increased translocation of HK2 to the outer mitochondrial membrane occurred on day 3 of MCA. The effect may improve mitochondrial coupling and reduce ROS production, thus preventing activation of apoptosis through opening of the MPT pore^37^. The HK2-mediated cardioprotective effect was documented under acute stress conditions such as pre-conditioning^38^ or severe chronic hypoxia^39^, but not under moderate regimens of chronic hypoxia^40^. It is noteworthy that PKG also prevents MPT pore opening via the activation of the mitoK(ATP) channel^41^. The presented data suggest differences in the cardioprotective targets in the early (3 days) and later stage (10 days) of MCA and indicate an important role for both PKG and HK2 in preservation of mitochondrial function during ischemia and reperfusion.

AMPK can be considered as another potential player in cold-induced cardioprotection in the present study, as after 10 days of MCA, the p-AMPK/AMPK ratio markedly increased. AMPK is known to exert pleotropic cytoprotective effects^42^ and can be activated by phosphorylation or allosterically when the increased energy expenditure leads to imbalance of the ATP/AMP ratio through adrenergic or thyroid system signaling, or by nutrient-specific upstream signals controlling cell survival and regeneration, and mitochondrial biogenesis^43^. The loss of AMPK sensitivity to activating stimuli is related to ageing^44^. Cardioprotective activation of the AMPK signaling pathway was reported in mice subjected to exercise, and this effect was absent in hearts of β3-AR knock-out mice^21,45^. Furthermore, both β3-AR and AMPK pathway prevents hypertrophic remodeling and fibrosis, while restoring the cellular energy balance^22,23,46,47^. Our findings align with the potential involvement of AMPK signaling in the protected cardiac phenotype induced by 10-day MCA without signs of hypertrophy.

### Batokines FGF21 and IL-6

Brown adipose tissue is regulated by adrenergic signaling and is a potential player in cold-elicited cardioprotective phenotype due to its endocrine function. We characterized BAT maturation based on a significant increase in the BAT/BW ratio, mitochondrial biogenesis, UCP1-dependent respiration, and altered FGF21 spatial expression. In humans, the cold-adaptive phenotype has a beneficial effect on obese and diabetic patients even after 10 days of intermittent cold exposure (14–15 °C for 10 consecutive days)^3,4^, which highlighting the clinical relevance of our model. The release of FGF21 from the liver and adipose tissue was reported to reduce cell death and to attenuate myocardial infarction in mice^48^. Additionally, it prevented hypertrophic stimuli via its anti-oxidant/anti-inflammatory action^49^. However, unlike in BAT, we did not observe increased FGF21 in serum, nor did we see changes in FGF21 spatial expression in heart tissue. Our finding suggests that FGF21 is unlikely to play a role in cardioprotection elicited by short-term MCA. Similarly, we can exclude a cardioprotective role of the IL-6 batokine, a key regulator of BAT growth, because its serum and heart tissue levels declined in both protective stages of MCA. Furthermore, acute ablation of BAT 2 h prior to the ischemic insult (considering 2-h half-life of FGF21), did not affect the infarct size-limiting effect (data not shown). Consequently, it appears unlikely that BAT plays a major role in the infarct size-limiting effect induced by MCA in our study. Current investigations into cold-therapy of diabetes, suggest that beneficial effects of CA may stem from tissues other than BAT, which is less abundant in humans^3^.

### Serum cytokines and chemokines

Moderate cold acclimation (MCA) exerts an impact on sympathetic nervous system, which is known to be a crucial regulator of immune responses especially during ischemic injury^15,50^. In the light of this, we have examined the effect of MCA on IL-6 and IL-10 in the LV cardiac tissue and conducted multiplex analyses of selected cytokines and chemokines in blood serum. We observed a significant reduction in IL-6 levels in the LV tissue. In the serum, MCA led to a decrease in pro-inflammatory Th1 cytokines (IL-17, IL-12p70, IL-6, IL-1α, IFNγ), and three anti-inflammatory Th2 cytokines (IL-4, IL-5, G-CSF). IL-17A participates in inflammation of blood vessels and cardiac cells and is also implicated in the pathogenesis of cardiovascular diseases that occur prematurely in chronic inflammatory disorders including atherosclerosis and myocardial infarction^51,52^. Likewise, while elevation of IL-6 is closely related to atherosclerosis, myocardial infarction, and heart failure, its transient increase also plays a role in tissue proliferation^53^. Activation of IFN-γ signaling pathways is thought to drive atherosclerosis, it is an important target for the prevention and treatment of cardiovascular diseases^54^. A marked decrease in IL-6 and IFN-γ corresponds to a decrease in heterodimeric IL-12p70, their direct regulator in innate adaptive responses^55^. Thus, the substantial downregulation of IL-17, IL-6 and IFN-γ suggests that short-term MCA mediates inflammation-suppressive immunomodulation that possesses a beneficial effect.

Of significant note, the calculated inflammatory ratios demonstrated that the balance between pro- and anti-inflammatory cytokines was maintained at equal level in the completely acclimated rats on day 10 of MCA. While the main players in Th1 pro-inflammatory responses were significantly suppressed by the MCA regimen, the chemotactic cytokines MCP-1 and MCP-3 were upregulated. MCP-1, monocyte chemotactic protein, contributes to routine immunological surveillance^56^, and MCP-3 was shown to stimulate the migration of circulating angiogenic cells and promote angiogenesis, suggesting its role in the cardiac repair processes^57^.

Regarding inflammatory responses in cardiac tissue, the decline in IL-6 indicates a moderate anti-inflammatory effect of MCA in the heart. It is known that acute I/R insult increases pro-inflammatory Th1 cytokines, as well as several chemokines in the heart tissue, levels of which are critical for subsequent cardiac remodeling and tissue repair^52,58,59^. In this context, the increased whole-body pro-inflammatory status observed during aging and various pathophysiological complications such as obesity and metabolic syndrome which often accompany cardiovascular diseases, might impair the healing process of the injured heart^60^. Moreover, acute coronary syndrome and atherosclerosis are accompanied by asignificant pro-inflammatory Th1/Th2 imbalance^61^. Therefore, we speculate that the MCA-elicited decline of predominantly pro-inflammatory cytokines, while maintaining the balance of the Th1/Th2 ratio, reduces the likelihood of an inflammation burst, thereby potentially contributing to the cardioprotective effect. Notably, the combination of cold exposure training with a breathing exercise robustly attenuates the inflammatory response in healthy young men^62^. However, repeated immersion in cold water (14° C for 1 h/day, 6 week) exhibited a slight stimulatory effect on the Th1-linked immune system of trained young men^63^. The given data suggests that the intensity and regimen of CA plays pivotal role in the immune response. Further in-depth studies are required in this area to fully comprehend the implications of the immune system in the context of MCA.

### Summary

The presented data provide a comprehensive overview of the impact of continuous exposure to moderate cold on β-adrenergic signaling in the left myocardium as well as a systemic profile of cytokines/chemokines during the acute and early phases of the exposure.

In this study we demonstrate that short-term moderate cold exposure enhances myocardial tolerance to I/R injury in rats within as early as 3 days without any apparent negative side effects such as hypertension or myocardial hypertrophy. The cold-elicited cardioprotective effect is accompanied by a reduction in the total number of adrenergic receptors in the membrane fraction, primary involving β1-ARs. Additionally, there is an attenuation of β2-ARs/Akt signaling and reinforcement of the minor subtype of β3-ARs/PKG/AMPK signaling. These observations suggest that moderate cold exposure leads to modulation of both stimulatory and inhibitory adrenergic pathways contributing to its cardioprotective effect. At the systemic level, our findings revealed a significant shift in the immune status after 10 days of moderate cold exposure implying an anti-inflammatory and immunosuppressive effect.

Taken together, short-term MCA is a safe, non-hypothermic intervention that stimulates endogenous protective pathways not only in the heart but also in the whole organism. This prepares the organism to better cope with acute oxygen deprivation. A detailed understanding of the underlying mechanisms of MCA is a prerequisite for its potential application in future clinical practice.

## Materials and methods

### Animals, cold exposure protocol, and ischemia–reperfusion injury

Male Wistar rats (12-week-old, 300–350 g body weight, specific-pathogen-free (SPF); from Velaz, s.r.o., Prague, Czech Republic; e.g. inclusion criteria for all experiments) were housed in pairs in well-bedded cages to minimize environmental and social stress. All experiments were performed in the “Winter-Spring” season (November till April) with 12/12 light/dark cycle. We took special care to minimize potential confounders. The animals had free access to water and standard diet (Altromin mod.1324, Velaz s.r.o). Rats were randomly divided into four groups. Three experimental groups were exposed continuously to 9 ± 1 °C for 1, 3 and 10 days (start and end of experiment was at 8 a.m.). The temperature was set below the threshold of shivering thermogenesis^28^. The control group was kept at 24 ± 1 °C throughout experiment. At the end of cold exposure, all animals were anesthetized (thiopental, 60 mg/kg; i.p.) at the respective temperature to avoid an acute thermoregulatory response. At the end of each experiment, hearts were excised under deep anesthesia for further analyses. The number of animals per group (“n”) and exclusion criteria are indicated in the respective methods for each experiment when applied.

### Cardiac ischemic tolerance

Anesthetized animals (n = 16 per group) were intubated and ventilated (Ugo Basile, Italy) at 60–70 strokes/min (tidal volume of 1.2 ml per 100 g of body weight). Blood pressure in the cannulated carotid artery and a single lead electrocardiogram were recorded using PowerLab and LabChart Pro software (ADInstruments, Australia). Left thoracotomy was performed as follows: a silk braided suture 5/0 (Chirmax s.r.o.) was placed around the left anterior descending coronary artery about 1–2 mm distal to its origin. After a 15-min stabilization, regional myocardial ischemia was induced by tightening the suture through a polyethylene tube. After a 20-min occlusion period, the ligature was released and the chest closed, air was removed from the thorax, and spontaneously breathing animals were maintained under deep anesthesia for 3 h. Then, hearts were excised and washed by perfusion with saline via the aorta. The area at risk (AR) was delineated by perfusion with 5% potassium permanganate when ligature was re-tightened and frozen at −20 °C as described^10,11,64^. Frozen hearts were cut into 1 mm thick slices and stained with 1% 2,3,5-triphenyltetrazolium chloride (Merck, pH 7.4 and 37 °C) for 30 min, then fixed by immersion in a to 4% paraformaldehyde solution^64^. After 3 days, both sides of the slices were photographed. The infarct size (IS), and the size of the AR and the left ventricle were determined using Graphic Cell Analyzer software^65^. Exclusion criteria for the I/R protocol included the occurrence of cardiac arrhythmias during the stabilization phase, animal death during the experiment, or unsuccessful staining. Based on the above criteria, four animals/hearts were excluded from each experimental group.

### Echocardiography

In a separate group of animals (n = 5), in vivo heart imaging was performed prior to and after the acclimation using multimodal Vevo3100/LAZR-X Imaging platform (FUJIFILM VisualSonics, Inc., Toronto, Canada) as follows. Anesthetized animals (isoflurane 3%, 1.2 l/min for initiation and 1.5% for maintenance of anesthesia; Baxter S.A.Bd, Belgium) were placed on a heating pad (up to 37 °C) and connected to electrodes for monitoring the ECG and respiration) using the MX201 transducer (15 MHz frequency). Heart dimensions and function were evaluated by means of the parasternal left ventricle (LV) long-axis view. Rat cardiology transducer and M-mode echocardiography were used (M-mode gain set to 50 dB, B-mode gain 30 dB). The transducer imaging range was set from 6 to 26 mm. the EKV mode acquisition and process style were standard, the frame rate was 1000 Hz, and PSLAX was pre-set. After monitoring, animals had a rest for 4 days to recover from anesthesia and were then exposed to MCA (9 °C) for 10 days, and the whole imaging procedure was repeated. The left ventricular stroke volume (LVSV), ejection fraction (LVEF), fractional shortening (LVFS) and cardiac output (CO) were evaluated using the Vevo lab software. No data were excluded from the analyses.

### Isolation of mitochondria

Hearts from another group of animals were excised from anesthetized rats (n = 6) (thiopental, 60 mg/kg) and briefly washed in ice-cold saline. The left and right ventricles and the septum were separated on ice. Immediately after that, interscapular BAT was isolated and properly cleaned from other tissues on ice-cold plate. Mitochondrial fractions were freshly isolated from both fresh LV and BAT as described previously^11,66^. The free LV and BAT tissues were homogenized at 0 ◦C by a Teflon–glass homogenizer as 10% and 5% homogenate, respectively in a medium containing 250 mM sucrose, 10 mM Tris/HCl, 2 mM EGTA and 0.5 mg/ml of fatty acid-free BSA, pH 7.2. The homogenate was centrifuged for 10 min at 600*g*, and the supernatant was centrifuged for 10 min at 10,000*g*. The mitochondrial sediment was washed twice in a sucrose medium without EGTA and BSA by centrifugation for 10 min at 10,000*g*. Pellets of washed mitochondria were re-suspended in 0.5 ml of 250 mM sucrose, 10 mM Tris/HCl, pH 7.2.

### Mitochondrial swelling—cardiac tissue

Fresh myocardial LV mitochondria were tested for calcium sensitivity using mitochondrial swelling as indicated by a decrease in absorbance at 520 nm, measured with a Perkin-Elmer Lambda spectrophotometer at 30 °C in a swelling medium (10 mM HEPES, 65 mM KCl, 125 mM sucrose, 5 mM succinate and 1 mM KH_2_PO_4_, pH 7.2) as previously described^11,66^. Briefly, isolated mitochondria (~ 0.4 mg of protein) were added to 1 ml of the medium to achieve absorbance of approximately 1. After 1 min of pre-incubation, the swelling medium containing 10 mM CaCl_2_ solution was added to reach a final concentration of 200 μM, and absorbance was read for 5 min at 1-s intervals. The maximum swelling rate obtained by deriving the swelling curve is expressed as the change in absorbance (ΔA520/1 s) and the moving average of the maximum rate was evaluated as a parameter of mitochondrial membrane permeability (MPT) pore stability. No data were excluded from the analyses.

### UCP-dependent respiration—brown adipose tissue

BAT mitochondria were assessed for UCP1-dependent respiration in freshly isolated samples using the O2k respirometer (Oroboros Instruments, Innsbruck, Austria). All assays were conducted in the K-medium (10 mM Tris, 80 mM KCl, 3 mM MgCl_2_, 5 mM KH_2_PO_4_, 0.5 mM EDTA) at 25 °C. UCP1-dependent respiration was directly evaluated by titration of substrates (5 mM pyruvate, 1.5 mM octanoyl-l-carnitine, 10 mM glutamate, 2 mM malate, 10 mM succinate) followed by titration of the UCP1 inhibitor ADP (10–15 mM) and the ATP synthase inhibitor oligomycin (2 µg/ml)^67^. No data were excluded from the analyses.

### Cardiac tissue fractionation

Hearts from another group of animals (n = 6) were excised after anesthesia (thiopental, 60 mg/kg) and briefly washed in ice-cold saline. The left and right ventricles and the septum were separated on ice within seconds and snap-frozen in liquid nitrogen, weighed, and stored in liquid nitrogen until use. The free walls of LVs were fractionated for further analyses of β-AR binding by radio-immunoassay and western blotting as follows^11,68^. Each frozen sample was placed in five volumes of ice-cold TMES buffer (20 mM Tris–HCl, 3 mM MgCl_2_, 1 mM EDTA, 250 mM sucrose; pH 7.4) containing protease and phosphatase inhibitors (cOMPLETE and PhosSTOP, Merck), cut into small pieces and homogenized on ice using the Ultra-Turrax (IKA, Germany) (24,000 rpm, 15 s), and followed by glass homogenizer with motor-driven Teflon pestle (1200 rpm, 2 min). Aliquots of each homogenate sample were stored in liquid nitrogen for further analyses. The homogenate was centrifuged (2100*g*, 10 min, 4 °C, Hettich Universal 320R; Hettich, Germany). The nuclear-free supernatant was collected, and the pellet homogenized in the same volume of TMES buffer and centrifuged again. The supernatants were combined and centrifuged (50,000*g*, 30 min, 4 °C, Beckman Optima L-90K, rotor Ti50, Beckman, USA). The pellet (crude membrane fraction) was homogenized in TMES buffer without sucrose, and aliquots were stored at −80 °C until use. Protein concentration was assessed using the Bradford method (Merck).

### β-Adrenoceptor binding radio-immunoassay

The total number of myocardial β-ARs was determined by the radioligand binding assay with the β-AR antagonist [3H]dihydroalprenolol ([3H]DHA) as described previously^11,69^. In brief, samples of the crude membrane fraction (containing 150 μg protein) were incubated in the medium (total volume of 0.2 ml) containing 50 mM Tris–HCl, 10 mM MgCl2 and 1 ascorbic acid at pH 7.4 along with the β-AR antagonist [3H]DHA (ARC, USA) in at decreasing concentrations from 6 to 0.19 nM. The incubation was carried out at 37 °C for 1 h. The reaction was terminated by adding 3 ml of ice-cold washing buffer (50 mM Tris–HCl, 10 mM MgCl_2_; pH 7.4), followed by filtration through a GF/C filter pre-soaked for 1 h with 0.3% polyethylenimine. The filter was washed twice with 3 ml of ice-cold washing buffer. After adding 4 ml of scintillation cocktail EcoLite (MP Biomedicals, USA), the radioactivity retained on the filter was assessed by liquid scintillation counting for 5 min. Non-specific binding (background signal) was defined as the signal that was not displaced by 10 μM l-propranolol, representing approximately 40% of the totally bound radioligand. Binding characteristics of β-ARs (Bmax and Kd) in the crude membrane fraction were calculated as previously described and statistically analyzed using One site-specific binding equation, in GraphPad Prism 9 software. All Bmax and Kd values obtained through nonlinear regression analysis of samples from individual animals were included. No exclusion criteria were applied to the results of individual animals. Addressing the original data processing, the following exclusion criteria were applied. For total and nonspecific binding, data points that did not fall on the curve of the nonlinear and linear regression analyzes, respectively, were subjected to review, and outliers that deviated from the mean by more than 25% were excluded.

### Western blot analysis

Individual LV (n = 5–6 per group) samples, diluted in Laemmli sample buffer (BioRad), of from the crude membrane fraction, nuclei-free supernatant and mitochondrial fraction from each group (20–30 µg, 20 µg and 30 µg protein per lane, respectively) were resolved by sodium dodecyl sulfate electrophoresis on 10–12% polyacrylamide gels at a constant voltage of 200 V using Mini-Protean Tetra Cell (Bio-Rad). The gel-resolved proteins were electro-transferred onto the nitrocellulose membrane (0.2 µm pore size, BioRad) at a constant voltage of 100 V and 350 mA current for 1 h using Mini Trans-Blot Module (Bio-Rad) according to manufactures instructions. The nitrocellulose membranes (0.22 μm pore, BioRad) were then blocked with 5% non-fat milk in Tris-buffered saline for 1 h, and incubated overnight at 4 °C with the following polyclonal antibodies, according to manufacturer’s instructions: β2-Ars (bs-0947R, Bioss, USA), β3-Ars (bs-1063R), Gs, Giα1/2, and Giα3 (RCS polyclonal antibody)^70^ for the crude membrane fraction, AMPK (2532, Cell Signaling, USA), p-AMPK^Thr172^ (sc-33524, Santa Cruz, USA), PKA (sc-365615), pPKA (sc-32968), PKG (C8A4, Cell Signaling) for the nuclei-free supernatant, and HK1 (sc-28885), HK2 (sc-6521) for the mitochondrial fraction. After washing, membranes were incubated with secondary HRP-conjugated anti-rabbit (A9169 or A0645, Merck), anti-mouse (sc-2371) or anti-goat antibody (AP180P). Protein bands were visualized with the ECL SuperSignal substrate (34075, Thermo Fisher Scientific, USA) using the LAS-4000 imaging system (Fujifilm, Japan). The intensity of protein bands was quantified densitometrically using Image J^71^. At least three samples from each group were run on the same gel in technical duplicates, quantified on the same membrane, and normalized to total protein content loaded per lane as determined by Ponceau S (Merck) staining. Prestained protein ladders (BioRad) were used as molecular weight markers. The accuracy and reproducibility of the chemiluminescence signal were validated by loading samples at ascending concentrations of 20 to 30 µg protein per lane. In the original data processing, exclusion criteria were sporadically applied when a normalized value of the triplicate in a gel differed by at least half an order of magnitude.

### Quantitative immunofluorescence microscopy

In separate animal groups (n = 5 per group), hearts were excised from anesthetized rats, relaxed by perfusion with the relaxation Tyrode solution and fixed with freshly prepared 4% formaldehyde solution using the Langendorff apparatus. LV samples were treated aspreviously reported^39,72^. Similarly, pieces of isolated BAT (1 mm^3^) were fixed with 4% formaldehyde, cryoprotected by 20% sucrose, frozen and stored at −80 °C. Sections (5–7 μm) of both BAT and LV samples were cut using a cryostat (Leica CM3050, Leica microsystems, Germany), rehydrated in PBS, permeabilized in ice-cold methanol, and incubated (5 min) in 1% SDS in PBS for antigen retrieval. Sections were incubated for 80 min in the blocking solution at room temperature (10% donkey serum, 10% goat serum, 0.3% Triton X-100, 0.3 M glycine in PBS), and incubated with the following primary antibodies at 4 °C overnight: UCP1 (ab-23841, Abcam, UK) and FGF21 (ab-171941) for BAT, β2-AR (bs-0947R), β3-AR (bs-1063R), HK1 (ab-150423), and HK2 (ab-78259) and PKG1 (C8A4, Cell Signaling) for the LV, and then with the secondary donkey anti-rabbit IgG AlexaFluor488 conjugate (A21206, ThermoFisher Scintific) for 1 h at room temperature. The fluorescence marker for mitochondria was the anti-OXPHOS Ab cocktail (ab-110412), while for sarcolemma, and T-tubules we used the wheat germ agglutinin (WGA) Alexa-647 conjugate (W-32466, ThermoFisher Scientific). Additionally, for the sarcoplasmic reticulum we used anti-phospholamban (ab-2865). Sections were mounted in ProLong Gold Antifade Reagent with DAPI (Invitrogen). Images were captured from at least 5 randomly selected fields of view (FOV) for each section using a wide-field inverted microscope (NikonTi2) equipped with a set of LED illumination for fluorescence imaging (Nikon, Tokyo, Japan). Images were deconcolved and pre-processed using the Nikon Microscope Imaging Software (NIS-Elements). The extent of co-localization was calculated using the thresholded Manders M1 or M2 coefficient^73^ through the Colocalization Threshold plug-in in FIJI software^74^. No data were excluded from the analyses and any missing values in the raw dataset were a result of the colocalization algorithm.

### Multiplex and ELISA analysis of blood serum and heart homogenate

Rat serum samples (n = 6 per group) were collected and stored in liquid nitrogen. Prior to analysis, they were thawed on ice and centrifuged (10,000*g*, 10 min, 4 °C) for pre-cleaning. Concentrations of 22 cytokines (G-CSF, GM-CSF, IFNγ, IL-1α, IL-1β, IL-2, IL-4, IL-5, IL-6, IL-10, IL-12p70, IL-13, IL-17A, TNFα) and chemokines (Eotaxin, GROα, IP-10, MCP-1, MCP-3, MIP-1α, MIP-2, RANTES) were analyzed using the Cytokine & Chemokine 22-Plex Rat ProcartaPlex™ Panel (EPX220-30122-901, Thermo Fisher Scientific). Serum samples were diluted at a 1:1 ratio with the Assay Diluent to minimize matrix effects. The same diluent was used as a blank and for the preparation of calibration standards. Reverse pipetting was employed for high accuracy in all liquid handling steps. All samples, standards and blanks were analyzed in two technical replicates following the manufacturer’s instructions. The fluorescence intensities of at least 100 beads per analyte were recorded using the Luminex 200™ analyzer with the xPonent software build 3.1.871.0 (Luminex Corp.) properly calibrated according to the manufacturer’s instructions. No data were excluded from the analyses. Sporadic missing values in the raw dataset were designated as *n.a.* outputs from the reader.

Raw data were processed in R statistical environment version 4.1.2^75^ using drLumi package^76^. The median fluorescence intensity (MFI) for Luminex xMAP data and absorbance for FGF21 ELISA data were used for standard curve fitting and quantitation of cytokine/chemokine concentrations as previously described^77^. Concentrations or MFI/absorbances of two technical replicates of each sample were averaged before further statistical analysis.

Concentration of FGF21 in rat blood serum and IL-6, and IL-10 in LV nuclear free supernatant were determined using an ELISA kit (ab-223589; BMS625, ThermoFisher Scientific, ab100764) following the manufacturer´s instructions with the following modifications: Serum was diluted 1:1 with Sample Diluent NS (ab-193972), and 850 μg and 425 μg of LV protein were loaded per well for IL-10 and IL-6 analyses, respectively. Kinetic evaluation of absorbance at 600 nm was performed for 20 min in 30 s intervals, with shaking between reads (Synergy microplate reader, Biotek, USA). After a 20-min interval, 100 µl of Stop Solution was added to each well and end-point absorbance at 450 nm was measured using the same instrument. No data were excluded from the analyses.

### Statistical analysis

The sample size of experimental animals was statistically estimated using the resource equation approach and adjusted in accordance with ethical standards for animal experimentation. For the analysis of infarct size, twelve hearts were included in each group. Mitochondrial fractions from half of the six LV samples were used for swelling analysis. In each group, six whole LVs were fractioned and used for WB analyses, ELISA, and the receptor binding assay. For quantitative immunofluorescence analysis, samples from five hearts per group were employed. Echocardiography involved five animals per group. Statistical analyses were conducted using the GraphPad Prism 8 software (GraphPad, San Diego, CA). The distribution of data was analyzed by Shapiro–Wilk and Kolmogorov–Smirnov normality tests. The identification of outliers was carried out using the ROUT (Robust regression and Outlier removal) method with a ROUT coefficient set at Q = 1%. For parametric data, one-way ANOVA with Dunnett’s multiple comparison test was employed to identify significant differences between the means of individual groups. The significance level of p ≤ 0.05 was considered statistically significant. Data are presented as means ± SD. To visualize the levels of analyzed cytokines and chemokines in six serum samples per group, the raw MFI values from Luminex measurement were log10 transformed. All values were further scaled and centered, and the heatmap was constructed using the R package ComplexHeatmap^78^. Using the same data matrix, principal component analysis (PCA) was also performed to identify the major contributors to differences among experimental groups. The first two principal components (PC1) and (PC2) were visualized using the R package ggbiplot^79^.

### Limitation of the study

It should be mentioned, however, that the smaller number of samples (n = 5) in some analyses could be considered a certain limitation of the study.

### Ethics statement

The study is reported in accordance with ARRIVE guidelines of animal research. Animals were handled in accordance with the Guide for the Care and Use of Laboratory Animals published by the US National Institutes of Health (NIH Publication, 8th edition, revised 2011). The experimental protocol was approved by the Animal Care and Use Committee of the Faculty of Science, Charles University and Ministry of Education, Youth and Sport, Prague, Czech Republic, No. MSMT-12457/2021-3.

## Data Availability

The datasets generated and analyzed during the current study are available in the FigShare public repository under the doi number https://doi.org/10.6084/m9.figshare.23301260.
